# Phenylbutyrate interferes with the Fanconi anemia and BRCA pathway and sensitizes head and neck cancer cells to cisplatin

**DOI:** 10.1186/1476-4598-7-24

**Published:** 2008-03-06

**Authors:** Kyunghee Burkitt, Mats Ljungman

**Affiliations:** 1 Department of Radiation Oncology, Division of Radiation Cancer Biology, University of Michigan Comprehensive Cancer Center, University of Michigan Medical School, Ann Arbor, MI 48109, USA; 2 Department of Environmental Health Sciences, Program in Toxicology, School of Public Health, University of Michigan, Ann Arbor, MI 48109, USA

## Abstract

**Background:**

Cisplatin has been widely used to treat head and neck cancer. One of the clinical limitations with this treatment, however, is that tumors that are initially responsive to cisplatin later acquire resistance. We have recently shown that a subset of head and neck cancer cell lines has a defective Fanconi anemia DNA damage response pathway and this defect correlates to cisplatin sensitivity. We have also shown that the histone deacetylase inhibitor phenylbutyrate sensitize human cells to cisplatin. In this study we explored whether phenylbutyrate may sensitize head and neck cancer cells by interfering with the Fanconi anemia pathway.

**Results:**

We found that the phenylbutyrate sensitizes head and neck cancer cell lines to cisplatin. This sensitization by phenylbutyrate correlated to a significant decrease in the formation of cisplatin-induced FANCD2 nuclear foci, which is a functional read out of the Fanconi anemia and BRCA (FA/BRCA) pathway. This abrogation of the FA/BRCA pathway by phenylbutyrate was not due to loss of FANCD2 monoubiquitylation but rather correlated to a phenylbutyrate-mediated reduction in the expression of the BRCA1 protein. Furthermore, we found that cancer cells defective in the FA pathway were also sensitized to cisplatin by phenylbutyrate suggesting that phenylbutyrate targets additional pathways.

**Conclusion:**

The results from this study suggest that phenylbutyrate may have therapeutic utility as a cisplatin sensitizer in head and neck cancer by inhibiting the FA/BRCA pathway through the down regulation of BRCA1 as well as by an FA/BRCA-independent mechanism.

## Background

Cisplatin is a widely used chemotherapeutic agent used against many different types of tumors [1,2]. However, the variable tumor responses limit the usefulness of cisplatin as a therapeutic agent. It has been shown that the variation in cisplatin response in ovarian cancer is linked to the status of the FA/BRCA pathway [3]. This pathway is involved in the processing of cisplatin-induced DNA damage and cells defective in the FA/BRCA pathway are hypersensitive to cisplatin and other agents that introduce interstrand DNA cross-links [4,5]. We recently showed that cisplatin sensitivity in head and neck cancer may also be linked to the FA/BRCA pathway since cisplatin-sensitive head and neck cancer cell lines were found to be defective in the formation of FANCD2 nuclear DNA repair foci [6]. This defect was corrected by exogenously expressing wild-type BRCA1 in these cells suggesting that attenuated expression or mutations of the BRCA1 gene may be responsible for the failure of the FA/BRCA pathway to launch an appropriate response in these cells which would explain their cisplatin hypersensitivity [6].

Cisplatin induces intrastrand DNA cross-links, which constitutes about 85–90% of all lesions, and interstrand DNA cross-links contributing about 1–2% to the total lesion burden [7-9]. It is thought that because of its high abundance, the intrastrand DNA cross-links may be the major class of lesions responsible for the toxic effects of cisplatin. However, due to its severe inhibiting effect on replication and transcription and the complicated nature of its repair, the lower yield-forming interstrand DNA cross-links may greatly contribute to the toxicity of cisplatin [7-9]. While intrastrand DNA cross-links are repaired primarily by the nucleotide excision repair pathway, interstrand DNA cross-links are repaired by a combination of repair enzymes from both nucleotide excision repair and homologous recombination [7]. In addition, translesion DNA synthesis polymerases [10,11] and the FA/BRCA pathway [3,12,13] contribute to the tolerance of interstrand cross-links although the mechanisms responsible for this protection are not understood in detail.

While cisplatin works well as a first-line therapy with an estimated 50% response rate, it is less effective if the tumor reoccurs [1]. As most tumors are heterogeneous, harboring cancer cells with a range of cisplatin sensitivities, cisplatin will preferentially kill off the cisplatin-sensitive cancer cells in the tumor while the surviving cisplatin-resistant cells will repopulate the tumor. This will make subsequent cisplatin treatments ineffective on reoccurring tumors [3]. Another drawback of cisplatin therapy is its dose-dependent toxicities. Thus, efforts are needed to explore whether there are agents that could be combined with cisplatin to overcome the cisplatin resistance of reoccurring tumors and to lower the doses of cisplatin needed for a therapeutic response.

We and others have previously shown that histone deacetylase (HDAC) inhibitors can sensitize human cells to cisplatin [14,15]. The mechanism for this sensitization is not clearly understood but may involve the down-regulation of the apoptosis antagonist Bcl-X_L _and the DNA double-strand break repair protein DNA-PK [16]. The HDAC inhibitor phenylbutyrate has shown a good clinical safety record when used to treat urea cycle disorders and cystic fibrosis [17-19]. Furthermore, laboratory studies have shown that phenylbutyrate has potential anti-tumor activity by specifically killing tumor cells [20] and by blocking the invasiveness of metastatic cancer cells [21].

In this study, we investigated whether phenylbutyrate could sensitize head and neck cancer cells to cisplatin. Our results show that three relatively cisplatin-resistant head and neck cancer cell lines were sensitized to cisplatin when they were pretreated with phenylbutyrate. The mechanism for sensitization may involve the abrogation of the FA/BRCA pathway since phenylbutyrate abrogated the formation of FANCD2 repair foci following cisplatin treatment and this abrogation correlated to a phenylbutyrate-mediated decrease in BRCA1 expression. In addition, phenylbutyrate sensitized one head and neck cancer cell line with a defective FA/BRCA1 pathway to cisplatin suggesting that phenylbutyrate targets multiple pathways that normally protect cells against cisplatin.

## Results

### Phenylbutyrate sensitizes head and neck cancer cells to cisplatin

**Figure 1:**
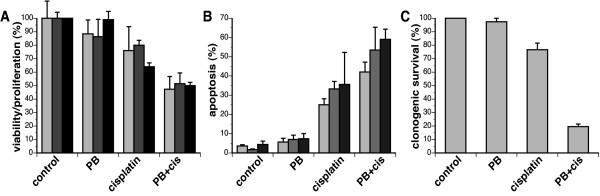
Phenylbutyrate sensitizes cisplatin-resistant head and neck cancer cell lines. (A) The head and neck cancer cell lines UM-SCC-1, -6 and -25 were treated with 2 mM phenylbutyrate (PB) for 5 days, 5 μM cisplatin for 3 days or pretreated with phenylbutyrate for 48 hrs followed by the addition of 5 μM cisplatin for 3 days. After treatments, cell viability/proliferation from each cell sample was determined using the WST-1 assay and values are expressed as a percentage of control cells. Error bars represent the standard error of the mean of triplicate samples. (B) Cells treated as in (A) were collected (both floating and attached cells), fixed and stained with propidium iodide and cells with sub-G1 DNA content were counted as apoptotic cells. The values are expressed as percentage of sub-G1 cells of total cells analyzed and error bars represent the standard error of the mean of triplicate samples. (C) Adherent UM-SCC-1 cells treated as in (A) were trypsinized and seeded at low densities in fresh media and incubated for 14 days. Clonogenic survival (%) is expressed as the fraction of surviving cells compared to control cells and expressed as a percentage with error bars represent the standard error of the mean of triplicate samples.

To investigate whether phenylbutyrate (PB) sensitizes head and neck cancer cells to cisplatin, three relative cisplatin-resistant head and neck cancer cell lines all expressing wild-type p53 (UM-SCC-1, -6, -25) were used. Cells were treated with 2 mM phenylbutyrate for 5 days, 5 μM cisplatin for 3 days or the combination of the two with cisplatin added on day three. The doses of phenylbutyrate and cisplatin used were chosen because they are clinically achievable. The effects these treatments had on the three cell lines were first analyzed by measuring viability/proliferation using the WST-1 assay. This assay is based on the reduction of the WST-1 reagent by viable cells to produces a soluble formazan salt that can be quantitated with an ELISA plate reader. Since the absorbance correlates with the number of viable cells in the sample, the readout is affected by cell death and inhibition of proliferation during the treatment period. When the three head and neck cancer cell lines were treated with either phenylbutyrate or cisplatin as single agents, viability/proliferation was reduced by 0–10% and 20–30%, respectively (Fig. 1A). When the two treatments were combined, the viability/proliferation was reduced by about 50%, which is more then an additive effect of each agent alone.

Since the WST-1 assay does not distinguish between loss of viability and inhibition of proliferation, we next investigated whether phenylbutyrate could sensitize the cells to cisplatin-induced apoptosis. Cells were treated as for the WST-1 assay described above, collected, fixed and stained with propidium iodide (PI) for the determination of apoptosis using flow cytometry. Treatment with phenylbutyrate alone only marginally increased the percentage of apoptotic cells over mock-treated cells while cisplatin treatment increased apoptosis by 20–30% over control cells (Fig. 1B). When cells were pretreated with phenylbutyrate for 48 hours before adding cisplatin, apoptosis was increased by 40–55% over mock-treated cells suggesting that the effect was more than additive.

Finally, we assessed the potential phenylbutyrate-induced sensitization of the cells to cisplatin using the clonogenic assay. This assay was only applicable to the UM-SCC-1 cell line since the other two cell lines failed to form colonies under our culture conditions. We found that phenylbutyrate did not significantly reduce the clonogenic survival of the UM-SCC-1 cells while cisplatin treatment reduced clonogenic survival by about 20%. When the two treatments were combined, we observed a much more dramatic cell kill than adding up the cell killing effects of the two drugs alone. Taken together, all three cell survival assays used suggest that phenylbutyrate sensitizes head and neck cancer cell lines to cisplatin.

### Phenylbutyrate attenuates the formation of cisplatin-induced FANCD2 nuclear foci

**Figure 2:**
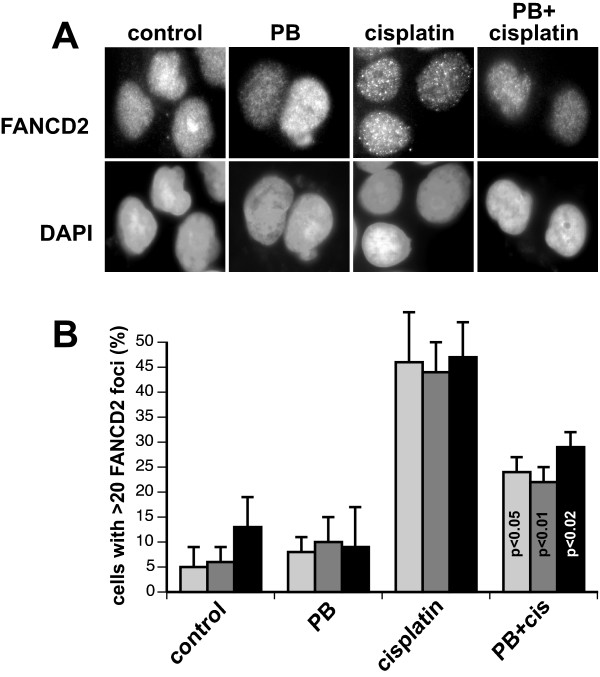
Phenylbutyrate abrogates the formation of FANCD2 foci following cisplatin treatment. (A) UM-SCC-6 cells were treated with 2 mM phenylbutyrate (PB) for 48 hours before adding 5 μM cisplatin and incubated for an additional 24 hours. Cells were then fixed and stained with anti-FANCD2 antibodies and the DNA dye DAPI. (B) The three head and neck cancer cell lines were treated as in (A) and FANCD2 foci were counted in a blind fashion using a fluorescence microscope. Cells containing more than 20 FANCD2 foci are expressed as a percentage of 100 cells analyzed for each condition and cell line. Error bars represents the standard error of the mean from three independent experiments. Statistical analysis using the student's t-test shows that phenylbutyrate significantly reduced the number of cells with > 20 FANCD2 nuclear foci. p-values are indicated in the graph. Bars: light gray, UM-SCC-1; gray, UM-SCC-6; black, UM-SCC-25.

What may be the mechanism by which phenylbutyrate sensitizes these head and neck cancer cell lines to cisplatin? Since cisplatin induces interstrand DNA cross-links and cells deficient in the FA/BRCA pathway are hypersensitive to cisplatin we next explored whether the cisplatin-sensitizing effect of phenylbutyrate correlated to a phenylbutyrate-mediated abrogation of the FA/BRCA pathway. The FA/BRCA response pathway is an important pathway that is thought to orchestrate the processing of DNA interstrand cross-links [4,5]. A functional readout of an activated FA/BRCA pathway is the formation of microscopic aggregates of FANCD2 proteins in nuclei, most likely representing sites of DNA damage. To investigate whether phenylbutyrate may interfere with the FA/BRCA response pathway and block the formation of FANCD2 nuclear foci after cisplatin treatment, the three different head and neck cancer cell lines were treated with phenylbutyrate, cisplatin or the combination of the two agents. Cells were then fixed and stained with anti-FANCD2 specific antibodies. Fluorescence microscopic inspection of the stained cells showed that cisplatin induced FANCD2 nuclear foci in about 45% of the cells analyzed while phenylbutyrate did not induce any FANCD2 foci over mock-treated control cells (Fig. 2). When the two agents were combined it was clear that phenylbutyrate significantly reduced the number of cells containing cisplatin-induced FANCD2 nuclear foci. Since phenylbutyrate did not affect viability or proliferation of the different cell lines (see Fig. 1A), we do not think the lowering of the number of cells with cisplatin-induced FANCD2 foci by phenylbutyrate is due to a redistribution of the cells in the cell cycle due to the activation of a cell cycle arrest. These results suggest that phenylbutyrate sensitizes head and neck cancer cells to cisplatin by abrogating the FA/BRCA pathway.

### Phenylbutyrate attenuates BRCA1 expression

**Figure 3:**
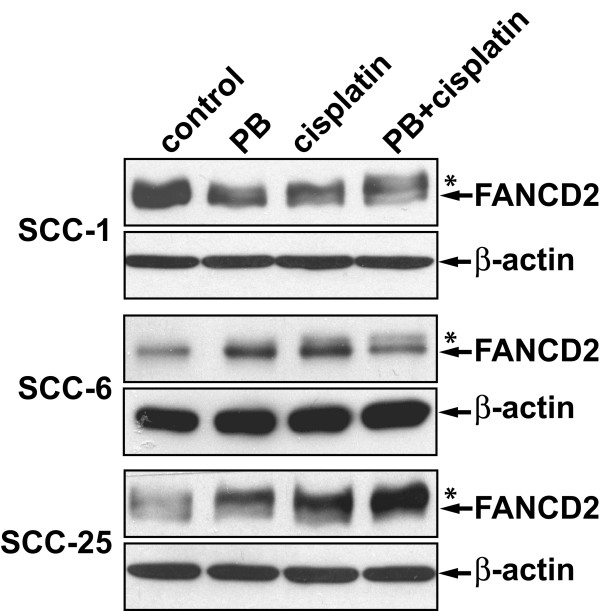
Cisplatin-induced monoubiquitylation of FANCD2 is not affected by phenylbutyrate in UM-SCC-1, -6 and -25 cell lines. Cells were treated with 2 mM phenylbutyrate (PB) for 48 hours before adding 5 μM cisplatin and incubated for an additional 24 hours. *represents the slower migrating monoubiquitylated form of FANCD2.

Because FANCD2 monoubiquitylation is required for the formation of FANCD2 foci, we next investigated whether phenylbutyrate may affect the cisplatin-induced monoubiquitylation of FANCD2. The three head and neck cancer cell lines were treated with phenylbutyrate, cisplatin or the combination of the two and the induction of monoubiquitylation of FANCD2 was evaluated using Western blot. It was found that cisplatin induced monoubiquitylation of FANCD2 regardless of whether the cells had been pretreated with phenylbutyrate or not (Fig 3). Thus, the phenylbutyrate-mediated abrogation of cisplatin-induced FANCD2 foci formation was not due to a loss of FANCD2 monoubiquitylation.

**Figure 4:**
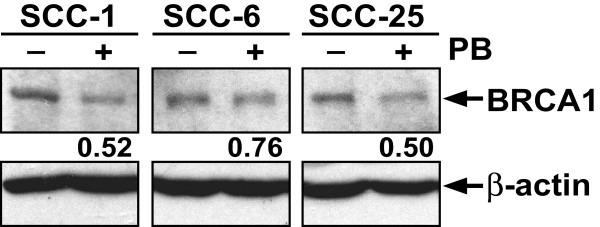
Phenylbutyrate down-regulates BRCA1 expression in head and neck cancer cell lines. Cells were treated with 2 mM phenylbutyrate (PB) for 3 days before the cells were harvested and the levels of BRCA1 protein analyzed with Western blot. The relative amount of BRC1 protein in the phenylbutyrate-treated samples is expressed as a fraction of the BRCA1 protein level in untreated controls.

It has been shown that in addition to monoubiquitylation the formation of FANCD2 nuclear foci following cisplatin treatment requires the tumor suppressor protein BRCA1 [22,23]. To test whether phenylbutyrate may affect the expression of BRCA1 in head and neck cancer cells, we analyzed the BRCA1 protein levels in mock-treated and phenylbutyrate-treated cells using Western blot. As can be seen in Figure 4A, phenylbutyrate reduced expression of BRCA1 by 48%, 24% and 50% in UM-SCC-1, 6 and 25, respectively. These results suggest that phenylbutyrate may sensitize head and neck cancer cells to cisplatin by interfering with the FA/BRCA pathway through the reduction of expression of BRCA1

### Phenylbutyrate sensitizes head and neck cancer cells by targeting multiple pathways

**Figure 5:**
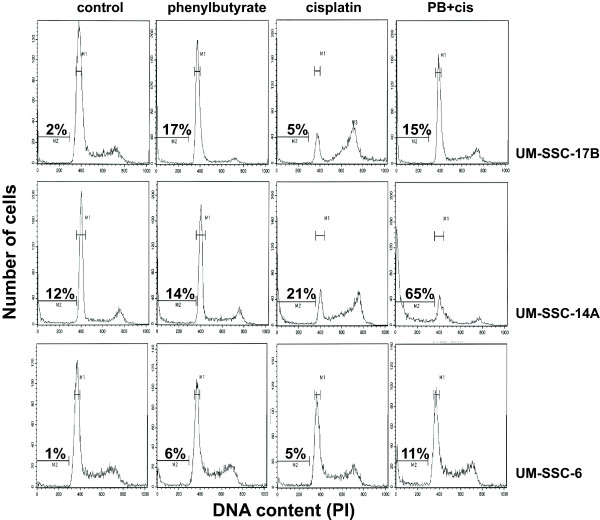
Phenylbutyrate acts on multiple targets to sensitize head and neck cancer cells to cisplatin. Top panel: BRCA1-defective UM-SSC-17B cells [6] were mock treated for 5 days (control), treated with 2 mM phenylbutyrate for 5 days (phenylbutyrate), treted with 2 μM cisplatin for 3 days (cisplatin) or treated with 2 mM phenylbutyrate for 5 days and 2 μM cisplatin for the last 3 days (PB+cis). At the completion of the treatment, both floating and attached cells were collected and analyzed by flow cytometry. Middle panel: same as above but using the FA/BRCA1-defective UM-SSC-14A cells [6]. Lower panel: Same as above but using the FA/BRCA1-proficient cell line UM-SSC-6 [6]. Note that the FA/BRCA1-defective cells appear to struggle through late S-phase as evidenced by gradual accumulation of cells in later stages of S-phase while the proficient UM-SSC-6 cell line do not show this accumulation. The percentages of sub-G_1 _DNA-containing cells (apoptotic cells) are presented in each panel.

If the cisplatin-sensitizing activity of phenylbutyrate is chiefly due to the inhibition of the FA/BRCA1 pathway, then phenylbutyrate should have only limited ability to sensitize head and neck cancer cell lines that are defective in the FA/BRCA1 pathway. To test this possibility, we pre-treated BRCA1-deficient UM-SSC-17B cells [6] with phenylbutyrate and then treated the cells with cisplatin. Since these cells are known to be sensitive to cisplatin we used a lower dose of 2 μM. Although the UM-SSC-17B cells were fairly sensitive to phenylbutyrate, no sensitization to cisplatin was apparent suggesting that phenylbutyrate may only sensitize cells with a functional FA/BRCA1 pathway (Fig. 5, top panels). However, when these experiments were performed using another head and neck cancer cell line (UM-SSC-14A) that also has a defect in the FA/BRCA1 pathway [6], a clear sensitization was observed (Fig. 5, middle panels). The percentage of apoptosis were 12% in controls, 14% for phenylbutyrate alone and 21% for cisplatin alone. When phenylbutyrate and cisplatin treatments were combined the amount of apoptosis rose to 65% which is clearly a more than additive effect. When the same treatment protocol was used on the FA/BRCA proficient cell line UM-SSC-6, we observed an additive effect of the two treatments. Since we used a lower dose of cisplatin in these experiments, the FA/BRCA-proficient cells are not as effected by cisplatin as shown in figure 1 where 5 μM cisplatin was used. It can be seen that both of the FA/BRCA1-defective cell lines show accumulation of cells in late S-phase and G2/M following cisplatin treatment which is in contrast to the similarly treated FA/BRCA1-proficient cell line UM-SSC-6. This is to be expected since the FA/BRCA pathway plays an important role in the coordination of processing of replication-blocking lesions. Taken together, the results suggest that phenylbutyrate do not inhibit the effectivness of cisplatin toxicity in cell lines with a defective FA/BRCA pathway which may have important clinical implications. Furthermore, phenylbutyrate may, in addition to inhibiting the FA/BRCA1 pathway, act on additional targets to sensitize head and neck cancer cells to cisplatin.

## Discussion

Head and neck and ovarian cancers are known to have heterogeneous clinical responses to the chemotherapeutic agent cisplatin [1,24,3]. A better understanding of this variable response as well as the development of novel strategies to sensitize resistant tumors to cisplatin would be of great importance for the clinical management of this disease. We have previously shown that the variability of the cisplatin response of a subset of head and neck cancer cell lines is linked to the functional status of the FA/BRCA pathway [6]. In this study we show that the HDAC inhibitor phenylbutyrate sensitizes cisplatin-resistant head and neck cancer cell lines to cisplatin. This sensitization appeared to be due to the abrogation of the FA/BRCA pathway by phenylbutyrate as well as through a FA/BRCA1-independent mechanism. Specifically, we show that phenylbutyrate inhibits the formation of FANCD2 nuclear foci after cisplatin treatment and this inhibition correlates to a down regulation of the tumor suppressor BRCA1.

There is currently great interest in HDAC inhibitors as anti-cancer agents [25,26]. The mechanisms for the anti-tumor activities of HDAC inhibitors include induction of apoptosis, cell cycle arrest, cell differentiation, and abrogation of tumor angiogenesis and invasion [26]. We have previously shown that the HDAC inhibitor phenylbutyrate down-regulates the anti-apoptosis protein Bcl-_XL _and the DNA-dependent protein kinase (DNA-PK) involved in double strand break repair and cellular stress signaling [16]. The consequence of the down-regulation of these proteins should lead to the lowering of the apoptotic threshold and inhibition of double strand break repair and thus phenylbutyrate and other HDAC inhibitors may have sensitizing properties when combined with radiotherapy or chemotherapeutic agents [26]. The results from this study concur with a sensitizing function of phenylbutyrate when combined with cisplatin. Measuring cell viability/proliferation, apoptosis and clonogenic survival revealed a more than additive effect when combining phenylbutyrate and cisplatin on head and neck cancer cell lines (Fig. 1). Thus, phenylbutyrate may be a useful agent to sensitize recurrent cisplatin-resistant head and neck tumors to cisplatin chemotherapy.

We recently showed that a subset of cisplatin-sensitive head and neck cancer cell lines are defective in cisplatin-mediated induction of FANCD2 nuclear foci [6]. In this study we show that phenylbutyrate abrogated the formation of FANCD2 nuclear foci following cisplatin treatment (Fig. 2). The formation of FANCD2 nuclear foci is thought to be essential for the proper processing of interstrand cross links during S-phase [4,5,27] and thus, the abrogation of FANCD2 foci formation by phenylbutyrate pretreatment is probably responsible for the cisplatin-sensitizing effect of phenylbutyrate in these cells. We also show that phenylbutyrate can sensitize FA/BRCA1-deficient head and neck cancer cells suggesting additional target for cisplatin sensitization by phenylbutyrate. It is possible that the cisplatin-sensitizing effect of phenylbutyrate is related to its role in targeting the expression of the apoptosis-antagonist Bcl-XL [16].

How does phenylbutyrate interfere with the formation of FANCD2 nuclear foci? A required step in order for FANCD2 proteins to form nuclear foci is that they become monoubiquitylated at the lys561 residue by the FA nuclear complex, consisting of at least eight different FA proteins [4,5,27]. When cisplatin-induced monoubuitylation of FANCD2 was analyzed for the three head and neck cancer cell lines, we did not observe any inhibiting effect of phenylbutyrate (Fig. 3). Thus, phenylbutyrate does not appear to interfere with the FA/BRCA pathway by inhibiting the monoubiquitylation of FANCD2. Another requirement for the formation of FANCD2 nuclear foci is that the cells harbor wild-type BRCA1 [23,22]. In a previous study we showed that cisplatin-sensitive cell lines having a non-functional FA/BRCA pathway were BRCA1 defective [6]. BRCA1-deficient cells are known to be hypersensitive to cisplatin [28-30] while BRCA1 over-expression has been shown to lead to increased resistance to cisplatin [31]. In this study we show that phenylbutyrate treatment leads to a down-regulation of BRCA1 in all three head and neck cancer cell lines tested (Fig. 4). Thus, the down-regulation of BRCA1 by phenylbutyrate may partially explain the abrogation of cisplatin-induced FANCD2 foci formation and the cellular sensitivity to cisplatin. We also show that phenylbutyrate must target other pathways in addition to the FA/BRCA1 pathway since the FA/BRCA1-defective cell line UM-SSC-14A was effectively sensitized to cisplatin by phenylbutyrate (Fig. 5).

Cisplatin is one of the most commonly used chemotherapeutic agents available today for the treatment of various malignancies [1,2]. However, its normal tissue toxicities, variable tumor responses and the selection for cisplatin-resistant cancer cells in reoccurring tumors limit the clinical usefulness of cisplatin. Recent efforts have been focused on screening for agents that sensitize tumor cells to cisplatin by inhibiting the FA/BRCA pathway [12]. One lead compound that interfered with cisplatin-induced FANCD2 monoubiquitylation and sensitized breast and ovarian cancer cells to cisplatin was the natural and relatively non-toxic compound curcumin. Our study identifies the HDAC inhibitor phenylbutyrate as an additional low toxicity agent that sensitizes cancer cells to cisplatin by interfering with the FA/BRCA pathway. Although further studies are needed to in more detail investigate the mechanisms responsible for the phenylbutyrate-induced abrogation of the FA/BRCA pathway, BRCA1 down-regulation and cisplatin-sensitization, our study opens up the possibility that phenylbutyrate could be used to sensitize cisplatin-resistant head and neck tumors in a clinical setting.

## Methods

### Cell lines and treatments

The head and neck cancer cell lines UM-SCC-1, -6, -25 were made available to us from the University of Michigan Head and Neck spore program. These cell lines were established from various anatomical locations of head and neck patients. Cisplatin-sensitivity based on the MTT assay has been previously assessed in these cell lines and ID_50 _for these cell lines were found to be 14.0, 36.7, 18.7 μM respectively [24]. The cells were grown in Dulbecco's modified Eagle's medium supplemented with 10% heat inactivated fetal bovine serum, penicillin/streptomycin in a humidified 5% CO_2 _incubator at 37°C. Cells were treated with 2 mM sodium phenylbutyrate (Scandinavian formulas, PA) for 48 hours and with 5 μM cisplatin (CDDP) (Sigma Chemicals, MO) and incubated for 72 hours.

### Cell proliferation Assay (WST-1 assay)

Exponentially growing UM-SCC-1, -6, -25 cell lines were plated in 96 well plates at a density of 10,000 cells per well and incubated in DMEM at 37°C overnight. Cells were then treated with 2 mM phenylbutyrate for 5 days, 5 μM cisplatin for 3 days or the combination of the two. At the completion of the 5 day incubation, 10 μl of cell proliferation reagent WST-1 (Roche, IN) was added into media in each well and the cells were incubated for 2 hr at 37°C. The absorbance (OD) of each well was determined with a spectrophotometer reading at a wavelength of 490 nm. Absorbance (OD) is assumed to be directly proportional to the number of viable cells.

### Flow cytometric analysis of apoptosis

Determination of the percentage of apoptosis induced following cisplatin treatment was performed as previously described [14,32]. Cells were treated with 2 mM phenylbutyrate for 5 days, 5 μM cisplatin for 3 days or the combination of the two. After incubation at 37°C, both floating and attached cells (trypsinized) were collected by centrifugation (1500 rpm for 5 minutes) and rinsed with PBS twice. To fix the cells, 500 μl of ice-cold 70% ethanol was added under mixing. After fixing cells for 30 minutes, cells were collected by centrifugation at 1500 rpm and rinsed with PBS twice. Cell pellets were resuspended in 500 μl of propidium iodide (PI) and incubated for 30 minutes at 4°C to stain cellular DNA. Cells with sub-G_1 _content of DNA were scored as apoptotic using flow cytometry (Coulter Elite ESP Cell sorter, FL) and the Multicycle software package (Phoenix Flow Systems, CA).

### Clonogenic survival assay

Cells were treated with 2 mM phenylbutyrate for 5 days, 5 μM cisplatin for 3 days or the combination of the two. At the completion of the 5 day incubation, cell were trypsinized and seeded in 60 mm plates at a low density (500 cells/dish) and cultured for 14 days in a humidified 5% CO_2 _incubator at 37°C. The cells were then rinsed with PBS and fixed and stained in a solution containing 0.25% crystal violet and 10% formalin (35% v/v) in 80% methanol for 15 minutes. Colonies were then counted and values are expressed as the fraction of cells surviving and normalized to the surviving fraction of control, which was set to a value of 100%.

### Immunoblotting

Cells were lysed with NP40 buffer (150 mM NaCl, 1% NP-40, 50 mM Tris (pH:8.0)), boiled for 5 min and subjected to 6% polyacrylamide SDS gel electrophoresis. After electrophoresis, proteins were transferred to Immobilon-P or Immobilon-FL transfer membranes (Millipore). The membranes were then blocked with 5% nonfat dried milk in TBS-T (50 mM Tris-HCl, [pH8.0], 150 mM NaCl, 0.1% Tween 20) and then incubated with primary antibodies diluted in Universal Antibody Buffer (30% BSA, 0.2% NaH_3_, 1% goat serum in TBS-T). The rabbit anti-FANCD2 antibodies (GeneTex, TX) were used in a 1:1000 dilution and the mouse anti-BRCA1 antibodies (Santa Cruz Biotechnology, CA) were used in a 1:100 dilution. After incubation with primary antibodies overnight at 4°C, membranes were washed with TBST and then incubated with secondary horseradish peroxidase-conjugated goat anti-rabbit or anti-mouse antibodies (Sigma, MO) at a 1:2000-fold dilution for 1 hr at room temperature. After rinses with TBS-T, immunoblotted proteins were captured on film by chemiluminescence.

### Immunofluorescence microscopy

Head and neck cancer cells were seeded on glass coverslips and incubated overnight before being treated with 2 mM PB for 3 days, 5 μM cisplatin for 24 hours or the combination of the two. Cells were then rinsed with PBS three times and fixed with 4% paraformaldehyde in PBS for 20 minutes at 4°C. The fixed cells were permeabilized with 2% Triton-X-100 in PBS for 8 minutes on ice. After blocking in Universal Antibody Buffer (30% BSA, 0.2% NaH_3_, 1% Goat serum in TBS-T) for 30 minutes at room temperature, anti-FANCD2 and/or anti-BRCA1 antibodies were added at dilutions of 1:200 and 1:100, respectively. After 1 hr incubation at 37°C, cells were washed three times with PBSBT (PBS, 0.5% BSA, 0.05% Tween 20) and then incubated with rabbit AlexarFluor555 (red) and/or mouse AlexaFluor 488 (green) for 1 hr at 37°C. After incubation, cells were rinsed with PBSBT three times. Glass coverslips were mounted in Vectashield containing DAPI. Images were captured on a Nikon microscope and processed using Adobe Photoshop software. All the quantification of FANCD2 foci was performed in a blind fashion.

## Abbreviations

BRCA1, breast cancer gene 1; FA, Fanconi anemia; HDAC, histone deacetylase inhibitor; PB, phenylbutyrate; UM-SCC, University of Michigan – squamous cell carcinoma.

## Competing interests

Part of this research project was funded by Virium Pharmaceuticals, Inc.

## Authors' contributions

KB participated in the design of the experiments, performed all the experiments and drafted the manuscript. ML designed experiments, prepared all the figures and wrote the final version of the manuscript. Both authors approved the final manuscript.

## References

[B1] Boulikas T, Vougiouka M (2004). Recent clinical trials using cisplatin, carboplatin and their combination chemotherapy drugs. Oncol Rep.

[B2] Kelland L (2007). The resurgence of platinum-based cancer chemotherapy. Nat Rev Cancer.

[B3] Taniguchi T, Tischkowitz M, Ameziane N, Hodgson SV, Mathew CG, Joenje H, Mok SC, D'Andrea AD (2003). Disruption of the Fanconi anemia-BRCA pathway in cisplatin-sensitive ovarian tumors. Nat Med.

[B4] D'Andrea AD, Grompe M (2003). The Fanconi anaemia BRCA pathway. Nat Rev Cancer.

[B5] Mirchandani KD, D'Andrea AD (2006). The Fanconi anemia/BRCA pathway: A coordinator of cross-link repair. Exp Cell Res.

[B6] Burkitt K, Ljungman M (2007). Compromised Fanconi anemia response due to BRCA1 deficiency in cisplatin-sensitive head and neck cancer cell lines. Cancer Lett.

[B7] Friedberg E, Walker G, Siede W, Wood R, Schultz R, Ellenberger T (2006). DNA Repair and Mutagenesis.

[B8] Siddik ZH (2003). Cisplatin: mode of cytotoxic action and molecular basis of resistance. Oncogene.

[B9] Wang D, Lippard SJ (2005). Cellular processing of platinum anticancer drugs. Nat Rev Drug Discov.

[B10] McHugh PJ, Sarkar S (2006). DNA Interstrand Cross-Link Repair in the Cell Cycle: A Critical Role for Polymerase zeta in G(1) Phase. Cell Cycle.

[B11] Albertella MR, Green CM, Lehmann AR, O'Connor MJ (2005). A role for polymerase eta in the cellular tolerance to cisplatin-induced damage. Cancer Res.

[B12] Chirnomas D, Taniguchi T, de la Vega M, Vaidya AP, Vasserman M, Hartman AR, Kennedy R, Foster R, Mahoney J, Seiden MV, D'Andrea AD (2006). Chemosensitization to cisplatin by inhibitors of the Fanconi anemia/BRCA pathway. Mol Cancer Ther.

[B13] Moynahan ME, Cui TY, Jasin M (2001). Homology-directed dna repair, mitomycin-c resistance, and chromosome stability is restored with correction of a Brca1 mutation. Cancer Res.

[B14] Chung DH, Zhang FF, Chen F, McLaughlin WP, Ljungman M (1998). Butyrate attenuates BCLXL expression in human fibroblasts and acts in synergy with ionizing radiation to induce apoptosis. Radiat Res.

[B15] Witzig TE, Timm M, Stenson M, Svingen PA, Kaufmann SH (2000). Induction of apoptosis in malignant B cells by phenylbutyrate or phenylacetate in combination with chemotherapeutic agents. Clin Cancer Res.

[B16] Goh M, Chen F, Paulsen MT, Yeager AM, Dyer ES, Ljungman M (2001). Phenylbutyrate attenuates the expression of Bcl-X-L, DNA-PK, caveolin-1, and VEGF in prostate cancer cells. Neoplasia.

[B17] Batshaw ML, MacArthur RB, Tuchman M (2001). Alternative pathway therapy for urea cycle disorders: twenty years later. J Pediatr.

[B18] Resar LMS, Segal JB, Fitzpatric LK, Friedmann A, Brusilow SW, Dover GJ (2002). Induction of fetal hemoglobin synthesis in children with sickle cell anemia on low-dose oral sodium phenylbutyrate therapy. J Pediat Hem Onc.

[B19] Rubenstein RC, Zeitlin PL (1998). A pilot clinical trial of oral sodium 4-phenylbutyrate (Buphenyl) in Delta F508-homozygous cystic fibrosis patients: Partial restoration of nasal epithelial CFTR function. Amer J Respir Crit Care Med.

[B20] Monneret C (2005). Histone deacetylase inhibitors. Eur J Med Chem.

[B21] Dyer ES, Paulsen MT, Markwart SM, Goh M, Livant DL, Ljungman M (2002). Phenylbutyrate inhibits the invasive properties of prostate and breast cancer cell lines in the sea urchin embryo basement membrane invasion assay. Int J Cancer.

[B22] Garcia-Higuera I, Taniguchi T, Ganesan S, Meyn MS, Timmers C, Hejna J, Grompe M, D'Andrea AD (2001). Interaction of the fanconi anemia proteins and BRCA1 in a common pathway. Mol Cell.

[B23] Vandenberg CJ, Gergely F, Ong CY, Pace P, Mallery DL, Hiom K, Patel KJ (2003). BRCA1-independent ubiquitination of FANCD2. Mol Cell.

[B24] Bradford CR, Zhu S, Ogawa H, Ogawa T, Ubell M, Narayan A, Johnson G, Wolf GT, Fisher SG, Carey TE (2003). P53 mutation correlates with cisplatin sensitivity in head and neck squamous cell carcinoma lines. Head Neck.

[B25] Petrie K, Prodromou N, Zelent A (2007). Histone deacetylase inhibitors in APL and beyond. Curr Top Microbiol Immunol.

[B26] Bolden JE, Peart MJ, Johnstone RW (2006). Anticancer activities of histone deacetylase inhibitors. Nat Rev Drug Discov.

[B27] Huang TT, D'Andrea AD (2006). Regulation of DNA repair by ubiquitylation. Nat Rev Mol Cell Biol.

[B28] Tassone P, Tagliaferri P, Perricelli A, Blotta S, Quaresima B, Martelli ML, Goel A, Barbieri V, Costanzo F, Boland CR, Venuta S (2003). BRCA1 expression modulates chemosensitivity of BRCA1-defective HCC1937 human breast cancer cells. Br J Cancer.

[B29] Quinn JE, James CR, Stewart GE, Mulligan JM, White P, Chang GK, Mullan PB, Johnston PG, Wilson RH, Harkin DP (2007). BRCA1 mRNA expression levels predict for overall survival in ovarian cancer after chemotherapy. Clin Cancer Res.

[B30] Quinn JE, Kennedy RD, Mullan PB, Gilmore PM, Carty M, Johnston PG, Harkin DP (2003). BRCA1 functions as a differential modulator of chemotherapy-induced apoptosis. Cancer Res.

[B31] Sylvain V, Lafarge S, Bignon YJ (2002). Dominant-negative activity of a Brca1 truncation mutant: effects on proliferation, tumorigenicity in vivo, and chemosensitivity in a mouse ovarian cancer cell line. Int J Oncol.

[B32] Ljungman M, Zhang F (1996). Blockage of RNA polymerase as a possible trigger for uv light-induced apoptosis. Oncogene.

